# A novel fuzzy system-based genetic algorithm for trajectory segment generation in urban global positioning system

**DOI:** 10.1016/j.jare.2025.06.007

**Published:** 2025-06-06

**Authors:** Xiaojuan Ran, Naret Suyaroj, Worawit Tepsan, Mu Lei, Hongjiang Ma, Xiangbing Zhou, Wu Deng

**Affiliations:** a International College of Digital Innovation, Chiang Mai University, Chiang Mai 50200, Thailand; b School of Information and Engineering, Sichuan Tourism University, Chengdu 610100, China; c School of Information and Engineering, Chengdu University, Chendu 610106, China; d School of Big Data and Artificial Intelligence, Yibin University, Yibin, 644000, China; e School of Electronic Information and Automation, Civil Aviation University of China, Tianjin, 300300, China; f Sichuan Provincial Key Laboratory of Philosophy and Social Sciences for Mountain Tourism Safety, Sichuan Tourism University, Chengdu 610100 China

**Keywords:** Trajectory generation, Fuzzy genetic algorithm, Automatic clustering, GPS data analysis, Least squares regression

## Abstract

**Introduction:**

The widespread adoption of Global Positioning System (GPS) technology has rendered trajectory data pivotal for urban traffic planning and travel pattern analysis. However, the traditional trajectory generation methods are constrained by manual cluster number settings, impeding both automation and optimality.

**Objectives:**

To address this issue, an enhanced Fuzzy System-based Genetic Algorithm (FGA) is proposed for automated trajectory segment generation.

**Methods:**

Based on the angle-based partitioning and cosine-constrained segmentation strategy, the proposed method integrates a fuzzy system into the genetic algorithm to dynamically adjust crossover and mutation probabilities. This enables automatic cluster number determination and sub-trajectory generation without human intervention. Moreover, the algorithm incorporates global search capability to avoid local optima. The FGA is further combined with least squares regression and applied to real-world taxi GPS data for trajectory reconstruction.

**Results:**

The experiment results demonstrate that FGA, when combine with different clustering algorithms (K-means, K-median, FCM), consistently identifies appropriate cluster numbers and produces globally optimal, smooth trajectory representations. The method improves clustering quality, trajectory continuity, and stability across multiple clustering strategies.

**Conclusion:**

The proposed FGA offers an effective and adaptive solution for trajectory segment generation in urban GPS system. Future work will explore enhancing its scalability, robustness to noise, and rule generalization across diverse datasets.

## Highlights

• Fuzzy-controlled dynamic adjustment of crossover/mutation in trajectory clustering evolution enables adaptive search strategies.
• Self-adapting trajectory segmentation via automatic cluster determination eliminates predefined parameter dependencies.
• Deep fuzzy-genetic fusion enables global trajectory optimization with feedback-driven adaptive regulation.
• Decoupled clustering-reconstruction framework uses least squares regression for smooth, interpretable trajectory continuity.

## Graphical abstract

**Figure:**
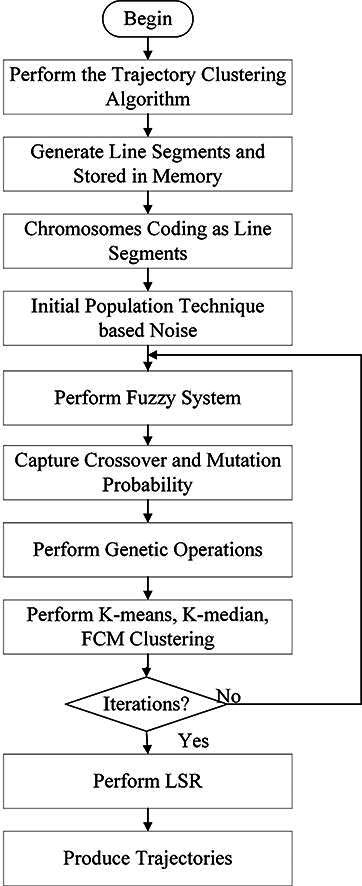

## Introduction

As the Global Positioning System (GPS) technology continues to advance and expand, GPS data has become an essential resource for analyzing urban traffic flows [[1], [2], [3], [4], [5]] and travel patterns [[6], [7], [8]]. These data not only act as the neural network of urban mobility by documenting the trajectories of moving objects [9,10], but also encapsulate essential urban information related to traffic conditions [[11], [12], [13]], travel demands [14,15], and population migration patterns [[16], [17], [18]]. In the fields of urban planning [19,20] and traffic management [[21], [22], [23], [24]], trajectory clustering technology offers valuable insights into vehicle movement patterns, thereby assisting urban planners and policymakers in optimizing traffic flow and mitigating congestion [25,26].

However, conventional trajectory generation methods often require manually determining of cluster numbers [[27], [28], [29]]. This approach not only complicates the analysis, but also hinders the automation and optimality of the clustering outcomes. The subjectivity inherent in manual parameter setting can undermine the effectiveness and reliability of the clustering results, making it challenging to adapt to the rapidly changing urban traffic environment.

To address aforementioned challenges, recent studies have increasingly explored intelligent optimization algorithms to enhance the adaptability and generalization of data modeling. particularly in image recognition and object detection, hybrid approaches such as GOA and PCA-KNN-based models [30,31] and multi-strategy fusion methods [[32], [33], [34], [35]] have achieved remarkable results in occlusion recognition and feature modeling tasks, which provide methodological insights for the structural optimization and intelligent evolution of trajectory clustering methods.

Building on these advancements, this paper proposes an enhanced Fuzzy-Genetic Algorithm (FGA) that combines fuzzy system with genetic optimization mechanism for adaptive urban trajectory segments. The algorithm focuses on solving critical issues such as automatic determination of the number of clusters and limited global search ability. By integrating the dynamic adjustment capabilities of fuzzy control systems with the global optimization strengths of genetic algorithms, the proposed FGA autonomously identifies the optimal number of clusters while effectively avoiding local optima. The algorithm initiates by generating trajectory fragments through angle-based partitioning, followed by population initialization using noise and density methods. During the clustering phase, it employs a multi-source similarity metric as its fitness function, dynamically adjusts crossover and mutation probabilities through fuzzy logic, and performs comprehensive global searches via genetic operators. Experimental results reveal that the FGA not only automates cluster number discovery but also achieves superior clustering performance when integrated with K-means, K-median, and FCM algorithms.

The innovations and main contributions of this paper are described as follows.

• **The First integration of fuzzy control systems** into trajectory clustering evolution processes, enabling intelligent search strategy adaptation through dynamic adjustment of crossover and mutation probabilities.
• **An automatic cluster determination mechanism** that self-adapts trajectory segmentation without requiring prior knowledge.
• **A deep fusion architecture** combining fuzzy systems with genetic search strategies, establishing an intelligent trajectory generation model with global search capability and feedback-driven regulation.
• **Decoupled processing framework** separating clustering from reconstruction phases, where least squares regression enhances trajectory continuity and system interpretability through smooth curve fitting.

This study advances trajectory generation methodology through four key innovations: (1) dynamic architecture design, (2) adaptive control mechanisms, (3) intelligent clustering strategies, and (4) optimized reconstruction processes. The proposed model effectively addresses critical limitations in conventional approaches, including structural rigidity, parameter inflexibility, and strong module coupling. Simultaneously, it demonstrates enhanced intelligent regulation, structural transparency, and adaptive performance in complex urban traffic scenarios.

## Literature review

### Traditional trajectory clustering methods

Trajectory generation plays a pivotal role in path planning and traffic flow analysis. The effectiveness of this process largely depends on efficient clustering algorithms, such as K-means and Fuzzy C-means (FCM). However, one significant limitation of these traditional methods is their dependence on a predefined number of clusters, which proves problematic in dynamic environments. As highlighted in Ref. [36], K-means and FCM struggle with determining the optimal number of clusters and selecting cluster centers due to the constraint of a fixed cluster count. This challenge is particularly evident in the complexity and variability of traffic trajectory data.

Ref. [33] introduces a clustering method for AIS data based on an unsupervised deep embedding framework. This method integrates autoencoders and deep clustering networks into a joint training process, enabling simultaneous representation learning and clustering optimization in a low-dimensional space. The approach demonstrates superior performance in feature extraction and modelling of complex structures, effectively identifying main ship channels. However, the clustering process is highly dependent on the predefined number of clusters, resulting in limited interpretability. Additionally, adapting this method directly to the complex environment of urban traffic trajectories poses significant challenges. In addition, Ref. [34] introduces Procrustes analysis into air traffic trajectory clustering to enhance feature representation and implement an automatic clustering scheme, providing a new approach for large-scale traffic data modelling. However, the method is dependent on specific application scenarios and cannot be directly extended to urban trajectory environments. It is evident that existing methods encounter limitations in terms of interpretability, cluster number presetting, and environmental adaptability. There is an urgent need for trajectory clustering frameworks that exhibit greater adaptability and generalisation ability.

### Intelligent optimization-based methods

Genetic Algorithm (GA) is widely applied in traffic trajectory generation and path planning, thanks to their superior global search capabilities. Through mechanisms such as selection, crossover, and mutation, GA effectively avoids local optima and iteratively refines trajectory outcomes. Nonetheless, conventional GA requires manual adjustment of key parameters, such as cluster numbers, limiting their adaptability in dynamic traffic scenarios.

Ref. [37] identifies limitations in traditional path planning algorithms, particularly their poor real-time responsiveness and adaptability in complex, high-density traffic conditions. In contrast, Ref. [38] explores the potential of genetic algorithms (GA) for calibrating lane-following behaviours using traffic data, noting GA's ability to improve computational efficiency and automation. Literature [39] outlines GA’s application in urban traffic route generation, emphasizing its success in static settings but noting limitations in dynamic environments due to fixed cluster numbers. In Ref. [40], an adaptive local trajectory planning method based on GA is proposed, which achieves real-time obstacle avoidance path adjustments by modulating optimization weighting factors. This method demonstrates adaptability in complex traffic settings.

Moreover, literature [41] introduces an enhanced GA for multi-objective optimization aimed at smoothing paths and minimizing turning angles. While it surpasses traditional GAs and A* algorithms in large-scale dynamic environments, further improvements in computational efficiency are needed. In [42], parking trajectory planning for onboard robots is optimized through polynomial parameterization combined with GA, although its efficiency diminishes in complex, dynamic environments. Lastly, Ref. [22] discusses an improved NSGA-II designed for conflict-free trajectory generation in high-density airspace, where the complexity of multi-objective optimization impacts convergence speed.

### Fuzzy and genetic approaches in trajectory modeling

Fuzzy logic, known for handling uncertainty, has been effectively used in clustering algorithms like the FCM. The FCM algorithm enhances data clustering by allowing data points to belong to multiple clusters with varying membership degrees. However, FCM, along with other fuzzy clustering algorithms, still requires presetting the number of clusters and is prone to local optima when processing large-scale or dynamic datasets, revealing its limitations. Ref. [43] proposed a fuzzy broad neuroevolution networks via multiobjective evolutionary algorithms. Ref. [44] proposed a deep learning model combining CNN and BiLSTM for trajectory time series prediction, which integrates features and a temporal attention mechanism to model the time series dependence of trajectory points, and employs a genetic algorithm for hyperparameter optimization. Although the approach achieves high predictive accuracy, it primarily focuses on future trajectory extrapolation in the temporal domain and does not address spatial segmentation or clustering of GPS trajectory data.

Ref. [45] focused on GPS trajectory spatial division and clustering, constructing a trajectory regression clustering method based on angle division, Hausdorff distance, and FCM clustering, with least square regression introduced to improve the trajectory continuity. While this method captures the structural features of trajectories effectively, it requires predefined cluster numbers and fixed genetic parameters, which limit its flexibility and adaptability to diverse data distributions. Similarly, Ref. [46] discusses an improved FCM algorithm for crowd-sourced lane-level map construction, which increases lane recognition accuracy but still faces challenges with large-scale urban traffic data. Ref. [47] used consensus clustering to subtype HCC based on SRGs and developed a method called signature-related gene analysis (SRGA) for identification of markers relevant to phenotype of interest.

Despite the promising results achieved by existing methods, limitations remain in cluster number dependency, rigid parameter configurations, and insufficient adaptability to complex urban trajectory environments. These challenges highlight the need for a more generalizable and adaptive clustering framework. To address these issues, this study proposes a fuzzy-genetic algorithm (FGA) that integrates fuzzy control into the genetic optimization process.

## Methodology

This section proposes a novel fuzzy-genetic algorithm (FGA) for trajectory segment clustering. In contrast to Ref. [33], which relies on deep embedding and fixed cluster settings, the proposed FGA adopts a fuzzy-controlled genetic optimization process that enables automatic cluster number determination and interpretable evolution. Compared to Ref. [43], which applies GA for hyperparameter tuning in temporal trajectory prediction, our method employs GA as the core clustering mechanism guided by fuzzy logic for spatial structure discovery. Unlike Ref. [44], which couples FCM with regression using pre-set parameters, our FGA dynamically adjusts crossover and mutation probabilities during evolution, improving adaptability and robustness. The following subsections detail the core principles and implementation of the proposed FGA.

### The concept of the FGA

**Fig. 1:**
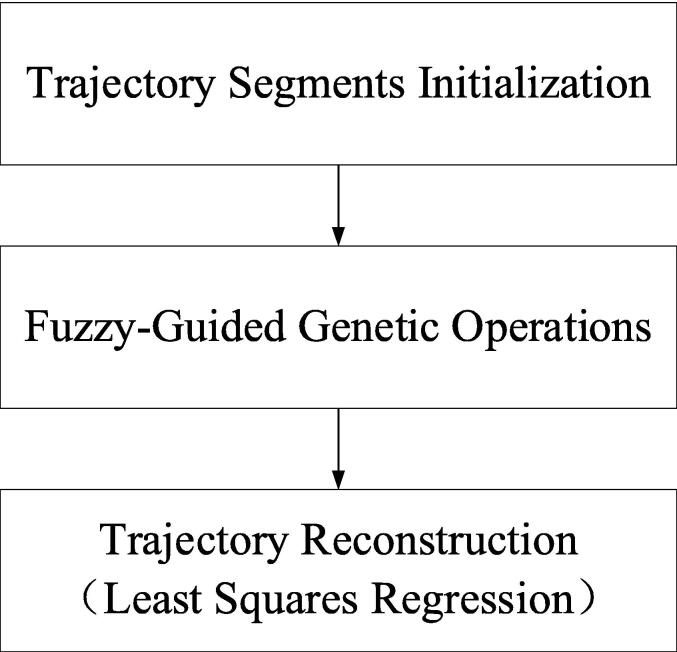
Simplified architecture of the proposed FGA framework.

The proposed FGA is structured into three key components, as illustrated in Fig. 1: trajectory segmentation initialization, fuzzy-guided genetic operations, and least squares-based trajectory reconstruction.

Int the first stage, raw GPS trajectories are segmented using similarity metrics [48] and Hausdorff distance [49] to extract meaningful sub-trajectory fragments that preserve spatial structural features.

The second stage introduces a fuzzy control mechanism into the genetic algorithm to dynamically adjust the crossover and mutation probabilities. This approach replaces traditional fixed or adaptively preset values. By fuzzifying genetic operations, the influence of raw fitness values on the search behaviour is decoupled, enhancing the algorithm’s global search capability and avoiding premature convergence.

In the final stage, the clustered trajectory segments are refined using Least Squares Regression (LSR) [46] to generate continuous and smooth trajectory paths.

#### The parameter settings of fuzzy system

To reduce the direct influence of fitness on genetic operations, this study introduces a fuzzy system that dynamically adjusts the crossover and mutation probabilities through its input parameters. Although the input parameters of the fuzzy system are determined by fitness values, this approach avoids the direct intervention of fitness in genetic operations, thereby generating more reasonable values for $P_{c}$ and $P_{m}$, and enhancing global search capabilities. Therefore, the Normalized Best Fitness (NBF) and Evolutionary Difference Indicator (DN), as defined in Equations (1), (2), are used as input variables to the fuzzy system. This design aims to address the tendency of chromosomes to fall into local optima during the evolutionary process and to overcome the limitations associated with fixed or improperly tuned crossover and mutation probabilities ($P_{c}$ and $P_{m}$) that may reduce search efficiency.

The normalized best fitness value (NBF) is calculated as follows:

(1) $$\mathrm{NBF}=\frac{f-f_{min}}{f_{max}-f_{min}}$$

where*, f* is the fitness value of the current individual,$f_{min}$ represents the estimated or actual minimum fitness value in the current population, and $f_{max}$ represents the fitness value that is equal to or greater than the maximum fitness value. NBF reflects the relative fitness level of an individual and serves as a basis for adjusting the operation intensity of genetic operators.

The evolutionary difference indicator (DN) is defined as:

(2) $$\mathrm{DN}=\frac{\left|\sum_{f_{\mathrm{PG}}-f_{\mathrm{CG}}<0}{(f}_{\mathrm{PG}}-f_{\mathrm{CG}})\right|}{N}$$

where, $f_{\mathrm{PG}}$ is the global best fitness from the previous generation, $f_{\mathrm{CG}}$ denotes the fitness values of individuals in the current population, and *N* is the population size. *DN* quantifies the average improvement of the current population relative to the previous global best and acts as a dynamic feedback signal within the fuzzy system for adaptively adjusting genetic operator probabilities. A larger DN indicates a substantial performance gap across the population, favoring a higher crossover and mutation probability to promote exploration. Conversely, a smaller DN implies convergence toward the optimum, warranting reduced variation to preserve high-quality solutions.

Unlike traditional parameter control strategies, such as fixed probabilities, linear annealing, or roulette-based heuristics—the proposed fuzzy control mechanism adaptively adjusts genetic operator probabilities based on NBF and DN, two real-time indicators of population state. This design enhances the responsiveness and robustness of the evolutionary process. Moreover, the use of normalized and population-sensitive metrics facilitates smooth integration into the fuzzy inference system, improving rule interpretability and control precision.

During the optimization process, the fuzzy control rules for crossover and mutation operations are defined as follows:

(1) It is essential that the chromosomes selected by the roulette participate in crossover as effectively as possible, ensuring that each chromosome has a fair opportunity to engage in crossover, which requires a higher crossover probability.
(2) To maintain population diversity after crossover, a randomly selected chromosome undergoes mutation. This operation generally requires a relatively lower mutation probability to avoid disrupting promising individuals.

Subsequently, based on the Mamdani fuzzy inference system and triangular membership functions, fuzzy rules are established to determine the crossover probability $P_{c}$ and mutation probability $P_{m}$, as shown in Table 1.

**Table 1:** Fuzzy rule sets for determining *P_c_* and *P_m_* from NBF and DN.

$P_{c}$		DN				$P_{m}$		DN			
		PS	PM	PB	PR			PS	PM	PB	PR
NBF	PS	PS	PM	PB	PB	NBF	PS	PS	PS	PS	PM
	PM	PM	PM	PB	PR		PM	PS	PM	PM	PM
	PB	PB	PB	PB	PR		PB	PB	PM	PB	PS
	PR	PB	PR	PR	PR		PR	PS	PM	PB	PS

**Note**: The table on the left defines the fuzzy rules for crossover probability $P_{c}$, and the table on the right defines those for mutation probability $P_{m}$. Each rule determines the output linguistic value of $P_{c}$ or $P_{m}$ based on the fuzzy input combinations of NBF and DN. Both input and output variables are represented by four fuzzy states—PS (small), PM (medium), PB (big), and PR (very big)—derived from triangular membership functions. In implementation, each linguistic value is mapped to a specific numerical value.

#### Principles of generation operations

The Genetic Algorithm (GA) is a heuristic intelligent optimization algorithm that mimics the biological process of selection, crossover, and mutation to iteratively generate improved solutions. It exhibits strong adaptability and global search optimization capabilities.

(1) Selection Operation

First, the fitness value of each chromosome in the initial population is calculated, and its initial seed point is recorded. The chromosomes are then sorted in descending order of their fitness values. Based on the problem requirements, the top *NIND* chromosomes are selected to form the selection population, denoted as ${POP}_{s}$, for subsequent genetic operations. The best chromosome is denoted as ${CR}_{best}$.

Next, the roulette wheel selection technique [43] is applied to select two chromosomes from the population for crossover. The selection probability based on the roulette wheel method is given by Equation (3):

(3) $$P\left({CR}_{i},{CR}_{j}\right)=\frac{f\left({CR}_{i},{CR}_{j}\right)}{\sum_{i=1,j=i+1}^{\mathrm{NIND}}f\left({CR}_{i},{CR}_{j}\right)}$$

where $f\left({CR}_{i},{CR}_{j}\right)$ represents the fitness value of the selected chromosome, *NIND* is the total number of selected chromosomes in the population, and the denominator is the sum of the fitness values of all chromosomes.

(2) Crossover Operation

In the crossover operation, two parent chromosomes generate two offspring chromosomes using a crossover operator and crossover probability. Common crossover operators include single-point crossover, two-point crossover, multi-point crossover, blend crossover and uniform crossover [44]. This study employs the single-point crossover operator, with the crossover probability $P_{c}$ automatically determined by the fuzzy system (as detailed in Section 3.1.1). Crossover occurs when $P_{c}$ exceeds the probability determined by the roulette wheel selection.

During the crossover process, a random breakpoint is selected on two parent chromosomes, and the gene segments following the breakpoint are exchanged to produce offspring chromosomes. After crossover, the length of the offspring chromosomes is checked to ensure it falls within the range $\left[2,\sqrt{n}\right]$. If this condition is met, the crossover is considered valid; otherwise, the process is repeated. However, when using the same breakpoint method, it is unnecessary to verify whether the offspring lengths lie within this range. Once a crossover operation is completed, the parent chromosomes are removed, and the process continues until all chromosomes have undergone crossover, resulting in a new crossover population ${POP}_{c}$.

The single-point crossover operation can be defined by Equation (4):

(4) $$SPC\left({CR}_{i},{CR}_{j},\alpha \right)=\left\{\begin{array}{c} {CR}_{i}^{\prime }=\alpha {CR}_{j}+\left(1-\alpha \right){CR}_{i}\\ {CR}_{j}^{\prime }=\alpha {CR}_{i}+\left(1-\alpha \right){CR}_{j} \end{array}\right)$$

where ${CR}_{i}$ and ${CR}_{j}$ represent the parent chromosomes, ${CR}_{i}^{\prime }$ and ${CR}_{j}^{\prime }$ represent the offspring chromosomes, and α is a random parameter that takes a value of 0 or 1. When α∈(0,1), it indicates that the probability of the parent chromosomes breaking at different positions is not equal.

(3) Mutation Operation

The mutation operation involves applying gene mutations to the chromosomes in the population to further explore the solution space and prevent the algorithm from getting trapped in local optima. In this study, chromosomes with low fitness values are selected for mutation to broaden the search space. Similar to the crossover operation, the mutation operation requires setting a mutation probability $P_{m}$, which is automatically determined by the fuzzy system (see Section 3.1.1).

The chromosomes are normalized according to Equation (5):

(5) $$R=\left\{\begin{array}{c} \frac{f-f_{min}}{f_{max}-f_{min}}\left(f_{max}>f\right)\\ 1(f_{max}>f_{min}) \end{array}\right)$$

where $f_{max}$ and $f_{min}$ represent the maximum and minimum fitness values in the population, respectively. For any given chromosome, the mutation value is generated within the range δ ∈ [−R,+R] and is scaled to the interval δ∈[-1,+1]. This distribution is used for gene mutation operations to introduce random variation, as defined by the mutation formula in Equation (6).

(6) $$f^{i^{\prime }}=\left\{\begin{array}{c} f^{i}+\delta \times \left(f_{max}^{i}-f^{i}\right)\\ f^{i}+\delta \times \left(f^{i}-f_{min}^{i}\right) \end{array}\right)$$

Here, $f_{max}^{i}$ and $f_{min}^{i}$ represent the maximum and minimum fitness values at the *i*-th gene of the chromosome during mutation,$f^{i}$ is the fitness value at the *i*-th gene before mutation, and $f^{i^{\prime }}$ is the fitness value after mutation. Once all chromosomes have undergone mutation, the mutated population ${POP}_{m}$ is generated, and the best chromosome ${CR}_{best}$ is identified.

#### Least squares regression in FGA

Least Squares Regression (LSR) is a well-established statistical technique, extensively applied in pattern recognition, data mining, and machine learning. It plays a crucial role in data classification, clustering, and trajectory regression [[50], [51], [52]]. LSR works by minimizing an objective function using a set of preprocessed data points to fit an empirical model or hyperplane. Specifically, it produces a hyperplane that best approximates the distribution of the data points.

This section examines the integration of LSR into the Fuzzy Genetic Algorithm (FGA) for unsupervised regression of clustering results, thereby generating smoother GPS trajectories. These optimized trajectories are invaluable for uncovering insights such as dynamic population movements [53] and traffic flow patterns [54,55] along the trajectories. Importantly, this regression process operates independently of map-based knowledge bases, yet it ensures that the number of smooth trajectories corresponds to the number of clusters.

Although LSR is not a core component of the FGA, it functions as an effective post-processing tool, significantly enhancing the smoothness and continuity of the generated trajectories. Incorporating LSR during the final stage of the algorithm or in subsequent applications improves both the algorithm’s practicality and its overall effectiveness.

The principle of Least Squares Regression (LSR) is to determine a set of regression coefficients that minimize the sum of squared residuals between the predicted and observed values [56]. The objective function is expressed as follows:

(7) $$\mathrm{MinimizeS}=\sum_{i=1}^{n}{\left(y_{i}-\sum_{k=1}^{m}A_{k}x_{i}^{k}\right)}^{2}$$

In this equation, *n* represents the number of observations,$y_{i}$ is the actual value of the *i*-*th* observation, $x_{i}^{k}$ denotes the *k-th* independent variable of the *i*-*th* observation, and $A_{k}$ represents the regression coefficients. The goal is to minimize the squared differences between the predicted and actual values, thereby producing smooth trajectories.

### Description and implementation of FGA

**Fig. 2:**
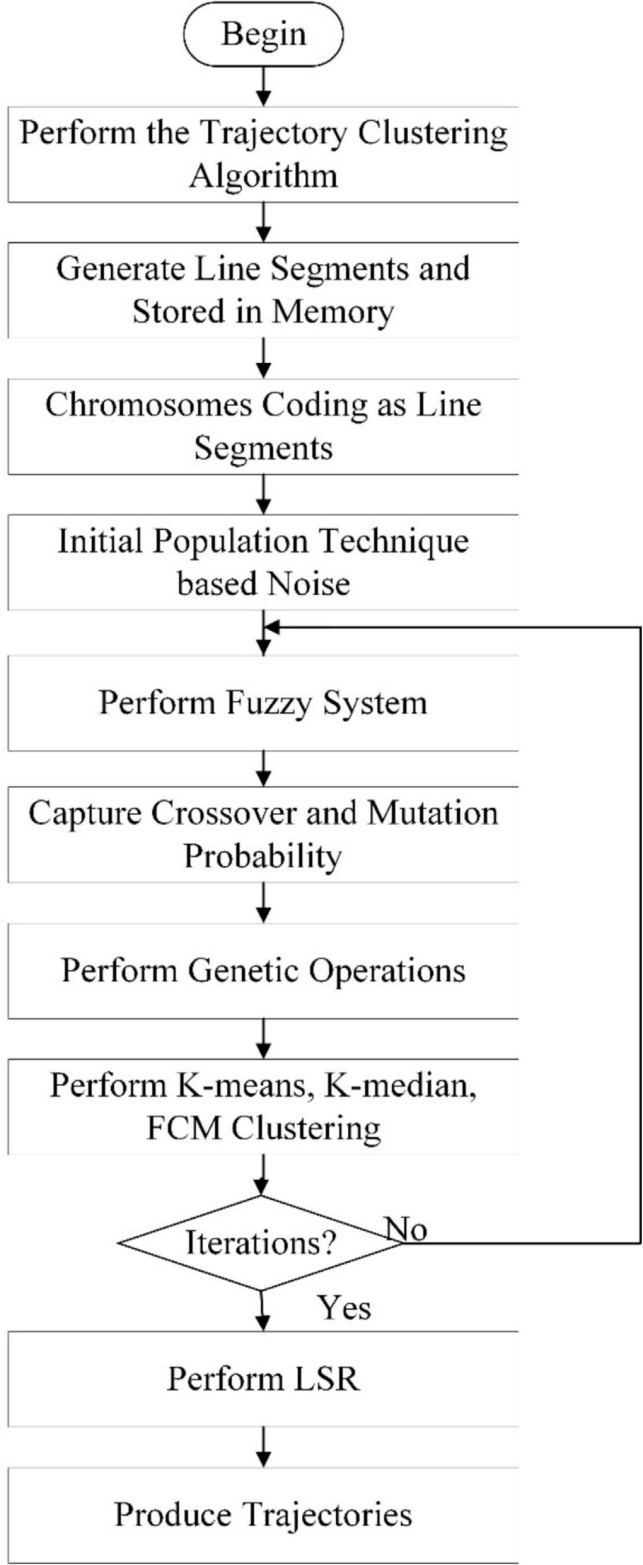
Overall workflow of the FGA algorithm.

The flow of the FGA algorithm is shown in Fig. 2, which provide a comprehensive overview of the entire process, from trajectory segment generation to the creation of smooth trajectories. The steps include generating trajectory segments, chromosome encoding, population initialization, fitness calculation, fuzzification, genetic operations, clustering, determining the conditions for iteration termination, and applying least squares regression to produce smooth trajectories.

A detailed explanation of the steps of the FGA algorithm is described as follows.

Step 1. Generating trajectory fragments

Utilize the angle-based partitioning and cosine-based constraint algorithm [39] to generate trajectory fragments.

Step 2. Chromosome encoding

Use the trajectory fragments for chromosome encoding in genetic operations. Each gene in a chromosome consists of three points, with multiple such genes forming a complete chromosome.

Step 3. Population initialization

Using the noise method from Ref. [52], population initialization is performed by replacing the maximum and minimum radii in the noise method with the noise radius from Equation (8).

(8) $$r_{\max}^{i}=1-\frac{r}{\mathrm{NIND}}\times i\left(i=1,2,\cdots ,NIND\right)$$

$$r_{\min}^{i}=r-\frac{r}{\mathrm{NIND}}\times i\left(i=1,2,\cdots ,NIND\right)$$

Where r is the sampling probability, as shown in Eq. (9), and *NIND* represents the population size:

(9) $$r=\frac{1}{\varepsilon^{2}N}$$

where ε is a user-defined parameter, and *N* represents the length of the dataset.

Step 4. Calculating fitness values

Use the similarity measurement Equation (10) [44], which incorporates multi-source information, to calculate the fitness values for genetic operations. This formula takes into account data from multiple sources, providing a more accurate assessment of individual differences:

(10) $$Sim\left(L_{j}\prime ,L_{j}\right)=\left\{\begin{array}{c} e^{\alpha l}\times \frac{e^{\beta h}-e^{-\beta h}}{e^{\beta h}+e^{-\beta h}}\;\;\;\;\;\;\;\;\;\;\;\;\;L_{j}\prime \neq L_{j}\\ 1\;\;\;\;\;\;\;\;\;\;\;\;\;\;\;\;\;\;\;\;\;\;\;\;\;\;\;\;\;\;\;\;\;\;\;\;\;\;\;\;other \end{array}\right)$$

where, α≥0 is a constant, β≥0 is a smoothing factor, $l=siml(L_{j}\prime ,L_{j})\;$ is the cosine similarity between $L_{j}\prime$ and $L_{j}$, and $h=min(dist\left(L_{j}\prime ,L_{j}\right))$ is the minimum Hausdorff distance between $L_{j}\prime$ and $L_{j}$.

Step 5. Fuzzification operation

Input the fitness values into the fuzzy system for fuzzification to generate crossover probability *P_c_* and mutation probability *P_m_* based on Table 1.

## Step 6. Genetic operations

Perform selection, crossover, and mutation operations using the probabilities generated by the fuzzy system.

Step 7. Clustering operation

Apply clustering techniques such as K-means, K-median, and FCM to further refine and optimize the population.

Step 8. Evaluating iteration termination conditions

Evaluate whether the iteration termination criteria have been met. If the conditions are satisfied, proceed to Step 9. Otherwise, return to Step 4 to continue the process.

Step 9. Producing smooth trajectories

Apply least squares regression (LSR) to generate smooth trajectories. LSR minimizes the sum of squared errors to produce smoother and more continuous trajectory paths.

## The experiment results and discussion

All experiments were conducted using MATLAB (version 2016b) on a system equipped with an Intel® Xeon® CPU E5-2658 (2 × 2.10 GHz), 32 GB of RAM, and Windows Server 2008, running in a VMware-based cloud environment. The clustering performance was evaluated using the silhouette coefficient (SC). In contrast to conventional methods based on Euclidean distance, this study adopts the Hausdorff distance for SC calculation to better capture the relative positional features of trajectory data. The parameter settings of the FGA are summarized in Table 2. To ensure the robustness and reliability of the results, each experiment was independently repeated 20 times under identical conditions.

**Table 2:** Parameter settings of the proposed FGA.

**FGA parameters**	**Setting**
Population Size	30
Maximum Iterations	60
PS	0–0.4
PM	0.2–0.6
PB	0.4–0.8
PR	0.6–1

### Description of the datasets

The GPS datasets utilized in this study were collected from three urban cities to ensure the generalizability and robustness of the proposed method [57,58]. The first dataset comprises records from approximately 30,000 taxis operating in a specific region of Beijing, China, collected during a ten-minute interval from 8:50 a.m. to 8:59 a.m. on March 20, 2016 [53]. The second dataset contains mobility traces of approximately 500 taxi cabs in the San Francisco Bay Area, USA, recorded over a 30-day period from May 17 to June 10, 2008 [59]. This dataset provides a long-term perspective on urban mobility patterns across varied spatial and temporal conditions. The third dataset, from Rome, Italy [60], recorded the GPS coordinates of approximately 320 taxis collected over a 30-day period from February 1 to March 2, 2014. Detailed dataset statistics are presented in Table 3. And Table 4 provides an example of field attributes from the Beijing dataset, including not only latitude and longitude but also additional information such as speed, angle, passenger status, and taxi operation status. These enriched data points contain valuable latent feature for trajectory analysis [[61], [62], [63], [64]] and pattern recognition [[65], [66], [67], [68]].

**Table 3:** Overview of the GPS datasets.

**Taxi GPS Dataset**	**Collection Area**	**Number of GPS Data Points**
Beijing (China)	0.20 × 0.30	71,375
San Francisco (USA)	0.50 × 0.50	54,556
Roma (Italy)	0.70*0.60	29,659

**Table 4:** Summary of Beijing taxi GPS dataset.

**Longitude**	**Latitude**	**Angle**	**Speed**	**Passenger status**	**Operating status**
40.03593	116.30856	69	0	Occupied	Doors closed, hybrid positioning, valid positioning, ACC on
40.05341	116.32346	327	977	Occupied	Doors closed, hybrid positioning, valid positioning, ACC on
40.03432	116.30051	159	565	Occupied	Doors closed, hybrid positioning, valid positioning, ACC on
40.04595	116.40405	24	0	Vacant	Doors closed, hybrid positioning, valid positioning, ACC on
39.95602	116.27868	267	0	Vacant	Doors closed, hybrid positioning, valid positioning, ACC on
40.04595	116.40405	24	0	Vacant	Doors closed, hybrid positioning, valid positioning, ACC on

### Clustering results and analysis

To comprehensively assess the effectiveness of the proposed FGA framework, experiments were conducted from three perspectives:

(1) Cluster generation behavior, indicated by the number of clusters automatically determined by the algorithms, which reflects the adaptiveness of the FGA model (see Table 5);

**Table 5:** Automatically generated cluster numbers using K-means, K-median, and FCM.

**Datasets**	**Algorithm**	**The number of clusters**			
		**Max**	**Min**	**Avg**	**Mode**
Beijing	K-means	13	10	10.9	11
	K-median	13	9	10.6	10
	FCM	12	9	10.7	12
San Francisco	K-means	9	6	7.45	7
	K-median	9	6	7.2	7
	FCM	13	10	11.4	11
Roma	K-means	8	6	6.6	7
	K-median	10	6	7.25	7
	FCM	14	10	11.9	12
(2) Clustering quality, evaluated using the Silhouette Coefficient (SC), which measures intra-cluster compactness and inter-cluster separation; higher SC values signify better-defined clusters (see Table 6);

**Table 6:** SC for clustering a trajectory segment using K-means, K-median, and FCM algorithms.

**Datasets**	**Algorithm**	**SC value**		
		**Max**	**Min**	**Avg**
Beijing	K-means	0.4518	0.4023	0.4284
	K-median	0.4663	0.4222	0.4396
	FCM	0.4559	0.3928	0.423
San Francisco	K-means	0.777	0.4368	0.7196
	K-median	0.7821	0.344	0.7028
	FCM	0.6735	0.5685	0.6296
Roma	K-means	0.6744	0.2936	0.5014
	K-median	0.7145	0.3648	0.5706
	FCM	0.4554	0.3406	0.4138
(3) Clustering validity, assessed via the PBM index, which evaluates the ratio of overall compactness to separation between clusters, serving as a complementary metric to SC for assessing the clustering structure, higher values indicate better-defined and more effective clustering structures (see Table 7).

**Table 7:** PBM index for clustering a trajectory segment using K-means, K-median, and FCM algorithms.

**Datasets**	**Algorithm**	**PBM value**		
		**Max**	**Min**	**Avg**
Beijing	K-means	0.0497	0.0402	0.0437
	K-median	0.0574	0.0384	0.0455
	FCM	0.0648	0.0382	0.0457
San Francisco	K-means	0.3027	0.1055	0.1799
	K-median	0.2538	0.1255	0.1838
	FCM	0.1888	0.0602	0.1338
Roma	K-means	0.2406	0.0832	0.1539
	K-median	0.2024	0.0867	0.1402
	FCM	0.0794	0.0317	0.0458

For trajectory clustering evaluation, the SC was computed using the Hausdorff distance rather than the traditional Euclidean distance, as it more effectively captures the spatial characteristics of trajectory data.

Table 5 shows the number of clusters automatically generated by the three algorithms under the FGA framework across all datasets. For the Beijing dataset, K-means and K-median yield average cluster numbers of 10.9 and 10.6, with modes of 11 and 10, respectively, indicating similar clustering behavior. FCM, on the other hand, has a slightly higher average of 10.7 and a mode of 12, reflecting its fuzzy partitioning mechanism that allows more flexible and fine-grained cluster boundaries. In the San Francisco dataset, all algorithms show high consistency, with averages cluster number of 7.45(K-means), 7.2(K-median), and 7.1 (FCM), and a unanimous mode of 7, suggesting a clearly defined cluster structure in the data. For the Rome dataset, FCM generates the highest average (11.9) and mode (12), along with a wider range (10–14), indicating finer partitioning but greater variability. K-means and K-median have lower averages (6.6 and 7.25) and modes at 7, showing stability but less ability to detect complex structures. Overall, FCM identifies more complex structures with variability, while K-means and K-median are more stable.

Building on the cluster numbers in Table 5, Table 6 evaluates the clustering quality using the SC, which reflects both cohesion and separation.

As shows in Table 6, no single clustering algorithm consistently surpasses the others across all datasets. Specifically, in Beijing, K-median achieves the highest SC value, indicating its superior robustness to noise and outliers in short-duration, high-density urban trajectories. In San Francisco, K-means demonstrates the best performance, reflecting its suitability for handling long-range sparse trajectories. However, in Roma, K-median again leads, suggesting that hard clustering methods may be more appropriate for this dataset's distribution.

These findings suggest that while FCM provides flexible cluster membership, this advantage does not universally translate into superior SC performance. The optimal choice of clustering algorithm depends on dataset characteristics, such as trajectory density, noise level, and spatial complexity.

To complement the SC analysis, the PBM index is adopted as an additional clustering validity measure. Table 7 reports the PBM values for all datasets, providing further insight into the clustering quality under the FGA framework.

In Table 7, the K-means algorithm consistently achieves the highest PBM values across datasets, indicating superior clustering performance in terms of compactness and separation. K-median follows closely, while FCM shows the lowest scores, particularly on the Roma dataset. This trend contrasts with the SC-based evaluation, where FCM often performs better. The discrepancy arises from the distinct emphases of the two metrics: PBM favors hard clustering with clearly defined boundaries, which aligns well with K-means and K-median, whereas FCM’s fuzzy membership mechanism allows overlap, leading to lower PBM scores despite its effectiveness in capturing complex structures.

It is important to note that the lower PBM values of FCM do not necessarily indicate weaker clustering performance. The proposed FGA framework incorporates a fuzzy control mechanism that conceptually aligns with FCM's soft clustering logic. This integration enhances adaptability to trajectory uncertainty and boundary ambiguity. Therefore, the reduced PBM scores primarily reflect the metric’s preference for crisp partitions, rather than an inherent deficiency in the FGA + FCM combination.

### Time complexity analysis of the FGA algorithm

In the FGA framework, clustering operations repeated throughout the evolutionary process constitute the primary computational burden. This section presents the theoretical time complexity of the three clustering algorithms and validates the analysis with empirical runtime results.

The K-means algorithm typically exhibits a time complexity as O(*nkt*), where n is the number of data points, k is the number of clusters, and t is the number of iterations. When *k* and t are treated as constants, the complexity simplifies to O(*n*), offering high efficiency for large-scale datasets.

The K-median algorithm follows a similar iterative structure but requires more computation to identify medoids, often approximated as O(*n^2^*), leading to moderate scalability within FGA.

The FCM has a higher complexity, approximately O(*nc^2^l*), due to fuzzy membership updates and cluster center recalculations in each iteration. Assuming l is small and constant, it reduces to O(*nc^2^*), making FCM the most computationally intensive among the three.

Table 8 presents the maximum, minimum, and average execution time of FGA integrated with K-means, K-median, and FCM across the three datasets.

**Table 8:** Average execution time of FGA with different clustering algorithms.

**Datasets**	**Clustering algorithm**	**Execution time (s)**		
		**Max**	**Min**	**Avg**
Beijing	FGA − K-means	5299.8	3256.5	4546
	FGA − K-median	4654.1	3464.3	4072.8
	FGA − FCM	5078.4	3711.1	4543.6
San Francisco	FGA − K-means	1139.7	553.4327	834.2845
	FGA − K-median	943.7282	360.8521	757.7137
	FGA − FCM	1223.1	722.3212	1043.5
Roma	FGA − K-means	2119	1137	1602.9
	FGA −K-median	2128.2	1158.5	1604.1
	FGA − FCM	2208	1485.7	1824.7

These results confirm the theoretical cost hierarchy: **O(FCM) > O(K-median) ≈ O(K-means).** Thus, in practice, clustering algorithm selection within FGA should consider the trade-off between computational efficiency and clustering quality, based on specific application requirements.

### Trajectory analysis using least squares regression

**Fig. 3:**
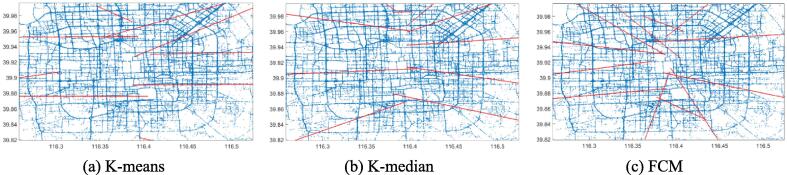
First-order LSR trajectories for K-means (a), K-median (b), and FCM (c) on the Beijing dataset.

**Fig. 4:**
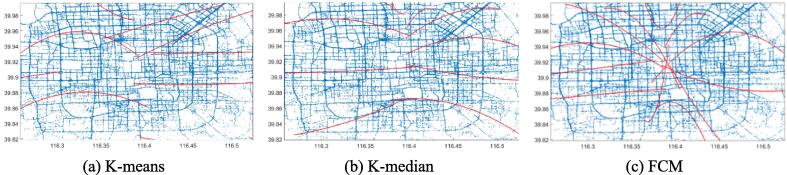
Second-order LSR trajectories for K-means (a), K-median (b), and FCM (c) on the Beijing dataset.

**Fig. 5:**
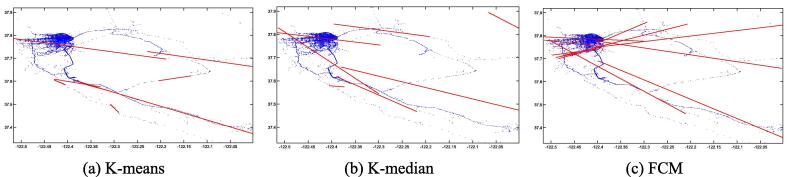
First-order LSR trajectories for K-means (a), K-median (b), and FCM (c) on the San Francisco dataset.

**Fig. 6:**
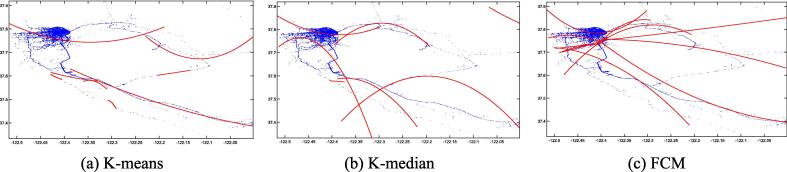
Second-order LSR trajectories for K-means (a), K-median (b), and FCM (c) on the San Francisco dataset.

**Fig. 7:**
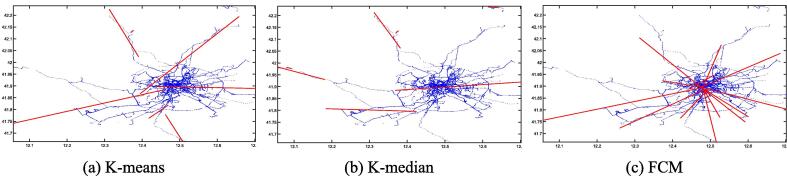
First-order LSR trajectories for K-means (a), K-median (b), and FCM (c) on the Roma dataset.

**Fig. 8:**
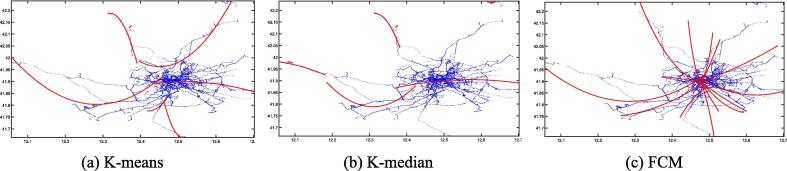
Second-order LSR trajectories for K-means (a), K-median (b), and FCM (c) on the Roma dataset.

This section presents the trajectory reconstruction results using least squares regression (LSR) based on clustering outputs from K-means, K-median, and FCM under the FGA framework. Fig. 3, Fig. 4, Fig. 5, Fig. 6, Fig. 7, Fig. 8 illustrate both first-order and second-order regression trajectories across three datasets.

Fig. 3, Fig. 4, Fig. 5, Fig. 6, Fig. 7, Fig. 8 illustrate that first-order regression produces straight-line approximations, which are computationally efficient but fail to capture the curvature of real-world roads. In contrast, second-order regression introduces quadratic terms, enabling smoother and more accurate trajectory fitting.

Among the clustering methods, FCM produces more balanced and representative cluster centers, resulting in more coherent regression trajectories with smoother, more natural boundaries. This advantage is particularly evident in regions with sharp turns or uneven sampling density, where FCM-based reconstructions align more closely with actual trajectories. In contrast, K-means and K-median exhibit greater deviations in such areas, leading to fragmented or unstable fits. Overall, the integration of FCM and second-order LSR within the proposed FGA framework offers superior trajectory reconstruction, particularly under noisy or structurally complex GPS conditions, demonstrating enhanced adaptability and robustness.

## Conclusion and future work

This paper presents a novel fuzzy system-based genetic algorithm (FGA) for automated trajectory segmentation and clustering. Experiments on real-world taxi GPS datasets from Beijing, San Francisco, and Roma validate the method’s effectiveness. By leveraging the spatiotemporal characteristics inherent in trajectory data, the proposed FGA framework integrates fuzzy membership modeling with adaptive genetic operator control. This approach enhances clustering flexibility and mitigates the instability associated with traditional fixed or fitness-dependent probability settings. The integration of fuzzy partitioning and genetic evolution enables automatic sub-trajectory generation and cluster number determination, providing valuable insights for urban road structure analysis and planning. Despite its effectiveness, several limitations should be acknowledged. First, the fuzzy rule base is currently designed manually, which may constrain adaptability across diverse datasets or application contexts. Second, as the population size and fuzzy system complexity increase, the algorithm’s computational cost may rise significantly, potentially limiting its real-time applicability. These limitations serve as important directions for future improvement.

Future research will focus on enhancing the scalability and efficiency of the proposed FGA framework for handling large-scale trajectory datasets. To address the time complexity, we plan to incorporate heuristic search strategies such as simulated annealing and tabu search, aiming to streamline the search process, reduce redundant operations, and accelerate convergence. Furthermore, we intend to integrate machine learning techniques to optimize the fuzzy membership functions by dynamically adjusting their parameters in response to data characteristics. In addition, future work will also explore the impact of different fuzzy rule configurations on clustering accuracy and stability, with the aim of establishing a more generalized and adaptive fuzzy rule optimization framework. These advancements are expected to improve the robustness, interpretability, and the adaptability of FGA, enabling more accurate and intelligent trajectory clustering for urban traffic management.

## Compliance with Ethics Requirements

This study does not contain any studies with human or animal subjects.

## CRediT authorship contribution statement

**Xiaojuan Ran:** Conceptualization, Methodology, Writing – original draft. **Naret Suyaroj:** Resources, Writing – review & editing. **Worawit Tepsan:** Conceptualization, Software. **Mu Lei:** Resources, Software. **Hongjiang Ma:** Writing – review & editing, Supervision. **Xiangbing Zhou:** Supervision, Funding acquisition. **Wu Deng:** Supervision, Funding acquisition, Resources, Writing – review & editing.

## Declaration of competing interest

The authors declare that they have no known competing financial interests or personal relationships that could have appeared to influence the work reported in this paper.
